# Genes Related to Frontonasal Malformations Are Regulated by miR-338-5p, miR-653-5p, and miR-374-5p in O9-1 Cells

**DOI:** 10.3390/jdb12030019

**Published:** 2024-07-06

**Authors:** Chihiro Iwaya, Sunny Yu, Junichi Iwata

**Affiliations:** 1 Department of Diagnostic & Biomedical Sciences, School of Dentistry, The University of Texas Health Science Center at Houston, Houston, TX 77054, USA; ciwaya@umich.edu (C.I.); sunny.yu@uth.tmc.edu (S.Y.); 2 Center for Craniofacial Research, The University of Texas Health Science Center at Houston, Houston, TX 77054, USA; 3 MD Anderson Cancer Center UTHealth Graduate School of Biomedical Sciences, Houston, TX 77030, USA

**Keywords:** microRNA, gene regulatory network, frontonasal malformations, craniofacial development

## Abstract

Frontonasal malformations are caused by a failure in the growth of the frontonasal prominence during development. Although genetic studies have identified genes that are crucial for frontonasal development, it remains largely unknown how these genes are regulated during this process. Here, we show that microRNAs, which are short non-coding RNAs capable of targeting their target mRNAs for degradation or silencing their expression, play a crucial role in the regulation of genes related to frontonasal development in mice. Using the Mouse Genome Informatics (MGI) database, we curated a total of 25 mouse genes related to frontonasal malformations, including frontonasal hypoplasia, frontonasal dysplasia, and hypotelorism. MicroRNAs regulating the expression of these genes were predicted through bioinformatic analysis. We then experimentally evaluated the top three candidate miRNAs (miR-338-5p, miR-653-5p, and miR-374c-5p) for their effect on cell proliferation and target gene regulation in O9-1 cells, a neural crest cell line. Overexpression of these miRNAs significantly inhibited cell proliferation, and the genes related to frontonasal malformations (*Alx1*, *Lrp2*, and *Sirt1* for miR-338-5p; *Alx1*, *Cdc42*, *Sirt1*, and *Zic2* for miR-374c-5p; and *Fgfr2*, *Pgap1*, *Rdh10*, *Sirt1*, and *Zic2* for miR-653-5p) were directly regulated by these miRNAs in a dose-dependent manner. Taken together, our results highlight miR-338-5p, miR-653-5p, and miR-374c-5p as pathogenic miRNAs related to the development of frontonasal malformations.

## 1. Introduction

Both genetic and environmental factors contribute to the etiology of frontonasal anomalies. Although whole-genome sequencing is commonly applied in medical research, the causes of nearly 70% of all birth defects in humans, including frontonasal anomalies, remain unknown. In addition, more than half of all birth defects involve craniofacial deformities, and the range of variation for any given facial trait often displays a substantial overlap between affected and healthy individuals [1].

The frontonasal prominence, which gives rise to the mid- and upper face, starts to develop with the thickening of the surface ectoderm in the frontonasal process at the 5th week of gestation in humans, and at embryonic day 10 (E10.0) in mice, and further develops into two bilateral nasal placodes, which form the lateral and medial nasal processes that are separated by the nasal pits at the center [2,3,4]. Frontonasal malformations including frontonasal hypoplasia, frontonasal dysplasia, and hypotelorism are caused by hypoplastic growth of the frontonasal prominence, and consequently of the nasal placodes [1,2]. Cranial neural crest (CNC) cells, which constitute the majority of the mesenchymal cells present in the craniofacial region that can give rise to various cell types [5], play a crucial role in the formation and growth of the frontonasal process [6]. To date, various genetic factors contributing to frontonasal malformations have been identified through mouse genetic studies; however, it remains elusive how epigenetic factors adversely influence gene expression during frontonasal development.

The development of the frontonasal region is regulated by a gene network, and a failure in this process results in frontonasal anomalies with varying severities. Although mild cases of frontonasal anomalies are harmless variations, in severe cases the condition is very impactful on both appearance and health, with effects such as obstructive sleep apnea, malocclusion, and dry eyes. Human and mouse genetic studies suggest that various genes are involved in frontonasal development [2]; however, the regulatory mechanisms of genes associated with frontonasal development remain largely unknown. Mice with loss of all mature microRNAs, which are short non-coding RNAs that regulate gene expression at the post-transcriptional level and fine-tune the expression of ~30% of all mammalian protein-encoding genes [7,8,9], in CNC cells (*Dicer^F/F^*;*Wnt1-Cre* mice) display severe frontonasal dysplasia [10,11,12], indicating that miRNAs play crucial roles in frontonasal development. Recent studies show that miRNAs are indeed essential for the survival of CNC cells during craniofacial development, and disruption of miRNA function in these cells results in frontonasal deformities in mice [3,13]; however, it is still largely unknown which and how miRNAs contribute to frontonasal development.

In this study, we aimed to identify miRNAs, and test their functional significance in the regulation of their downstream genes, that contribute to the pathogenesis of frontonasal malformations.

## 2. Materials and Methods

### 2.1. Gene Search

To identify a set of genes related to frontonasal malformations in mice, we searched the Mouse Genomic Informatics (MGI) database (https://www.informatics.jax.org, 1 February 2024) using ‘frontonasal hypoplasia’, ‘frontonasal dysplasia’, and ‘hypotelorism’ as search terms. All genes were further evaluated from the referenced literature for validation purposes.

### 2.2. Bioinformatic Analysis

miRNA target gene regulatory mechanisms were predicted using miRTarbase, miRanda, PITA, and TargetScan, with the Fisher’s exact test for determining the significance level of the shared genes between miRNA targets and genes related to mouse frontonasal malformations, as previously described [14]. The Benjamini–Hochberg procedure was used for multiple test correction. A Kyoto Encyclopedia of Genes and Genomes (KEGG) pathway analysis (http://www.genome.jp/kegg, 1 February 2024) was conducted to identify shared biological system(s) with ShinyGO ver0.80 [15,16,17]. A Gene Ontology (GO) enrichment analysis (http://www.geneontology.org, 1 February 2024) was conducted using the ShinyGO ver0.80 [15] for cell component (CC), biological process (BP), and molecular function (MF). Significantly enriched categories for the genes were filtered with a false discovery rate (FDR)-adjusted *p*-value < 0.05 using the hypergeometric test and at least four genes related to frontonasal malformations. Hierarchical level 4 was used at the cut-off in order to avoid too general GO terms.

### 2.3. Cell Culture

O9-1 cells, a neural crest cell line (SCC049, Millipore Sigma, Burlington, MA, USA), were maintained in medium for embryonic stem cells (ES-101-B, Millipore Sigma) at 37 °C in a humidified atmosphere with 5% CO_2_, as previously described [18].

### 2.4. Cell Proliferation Assay

O9-1 cells were treated with a mimic for the negative control (4464061; mirVana miRNA mimic, ThermoFisher Scientific, Waltham, MA, USA), miR-338-5p, miR-653-5p, or miR-374c-5p (4464066; mirVana miRNA mimic), or an inhibitor for the negative control (4464079; mirVana miRNA mimic), miR-338-5p, miR-653-5p, or miR-374c-5p (4464084; mirVana miRNA inhibitor), using the Lipofectamine RNAiMAX transfection reagent (ThermoFisher Scientific) according to the manufacturer’s protocol. Cell proliferation was measured using the Cell Counting Kit 8 (Dojindo Molecular Technologies, Inc., Kumamoto, Japan) 24, 48, or 72 h after each treatment (*n* = 6 per group), as previously described [18].

### 2.5. Bromodeoxyuridine (BrdU) Incorporation Assay

O9-1 cells were plated onto 35 mm dishes at a density of 10,000/dish and treated with a mimic for a negative control (4464061; mirVana miRNA mimic, ThermoFisher Scientific), miR-338-5p, miR-374c-5p, or miR-653-5p (4464066; mirVana miRNA mimic), using the Lipofectamine RNAiMAX transfection reagent (ThermoFisher Scientific) according to the manufacturer’s protocol. After 72 h, the cells were incubated with BrdU for 1 h. Incorporated BrdU was stained with a rat monoclonal antibody against BrdU (ab6326; Abcam, 1:1000), as previously described [19]. A total of ten fields, which were randomly selected from three independent experiments, were used for the quantification of BrdU-positive cells. Hematoxylin was used for counter staining. Color images were taken with a light microscope (BX43, Olympus, Tokyo, Japan).

### 2.6. Immunocytochemical Analysis

O9-1 cells were plated onto 35 mm glass-bottom dishes at a density of 10,000/dish and treated with a mimic for a negative control (4464061; mirVana miRNA mimic, ThermoFisher Scientific), miR-338-5p, miR-374c-5p, or miR-653-5p (4464066; mirVana miRNA mimic), using the Lipofectamine RNAiMAX transfection reagent (ThermoFisher Scientific) according to the manufacturer’s protocol. The immunocytochemical analysis was performed as previously described [19], using rabbit monoclonal antibodies against Ki-67 (ab16667, Abcam, 1:600). Hematoxylin was used for counter staining. Color images were taken under a light microscope (BX43, Olympus).

### 2.7. Terminal 2′-Deoxyuridine, 5′-Triphosphate (dUTP) Nick-End Labeling (TUNEL) Staining

O9-1 cells were plated onto 35 mm dishes at a density of 10,000/dish and treated with a mimic for a negative control (4464061; mirVana miRNA mimic, ThermoFisher Scientific), miR-338-5p, miR-374c-5p, or miR-653-5p (4464066; mirVana miRNA mimic), using the Lipofectamine RNAiMAX transfection reagent (ThermoFisher Scientific) according to the manufacturer’s protocol. The Click-iT Plus TUNEL Assay with Alexa 594 (C10618, Molecular Probes) was used to detect apoptotic cells, as previously described [20]. A total of four fields, which were randomly selected from two independent experiments, were used for the quantification of TUNEL-positive cells. Images were taken with a confocal microscope (Ti-E, Nikon, Tokyo, Japan).

### 2.8. Quantitative RT-PCR

O9-1 cells were treated with either mimic or inhibitor for miR-338-5p, miR-653-5p, miR-374c-5p, or negative control at 80% confluence, as previously described [18]. Twenty-four hours after the transfection, total RNA was extracted with the QIAshredder and miRNeasy Mini Kit (QIAGEN, Hilden, Germany), according to the manufacturer’s protocol (*n* = 6 per group). Extracted total RNAs were converted to cDNA, and gene expression was analyzed with quantitative RT-PCR (qRT-PCR) using the CFX96 Touch Real-Time PCR Detection system (BioRad). The PCR primers used in this study are listed in Supplementary Table S1. The expression of each gene was normalized with *Gapdh* expression. The miRNA expression was measured with TaqMan Fast Advanced Master Mix and TaqMan Advanced miRNA cDNA Synthesis Kit (ThermoFisher Scientific) or All-in-One miRNA qRT-PCR Reagents Kits (GeneCopoeia, Rockville, MD, USA), according to the manufacturer’s protocol.

### 2.9. Taqmann Assay

miRNA expression was measured in the frontonasal primordium of C57BL/6J mice at E10.5, E11.5 and E12.5, using the Taqman Fast Advanced Master Mix and Taqman Advanced miR cDNA Synthesis Kit (Thermo Fisher Scientific), according to the manufacturer’s instructions. Probes for miR-224-3p (mmu481009_mir), miR-383-3p (mmu481150_mir), miR-6951-3p (mmu482850_mir), and miR-7116-3p (466435_mat) were obtained from Thermo Fisher Scientific. The expression was normalized with U6 (4427975).

### 2.10. Statistical Analysis

Statistical analysis between two groups was performed with a two-tailed non-parametric Student’s *t*-test. Multiple comparisons were conducted with one-way analysis of variance (ANOVA) with the Tukey–Kramer post hoc test. Cell proliferation assays were analyzed with a two-way ANOVA. All results were obtained from three independent experiments; all experimental data were analyzed using the Prism software (GraphPad Software, Prism 10.1.2). A *p*-value < 0.05 was considered statistically significant. For all graphs, the data are represented as mean ± standard deviation (SD) with *n* = 6 per group.

## 3. Results

### 3.1. Identification of a Set of Genes Related to Frontonasal Malformations

**Figure 1:**
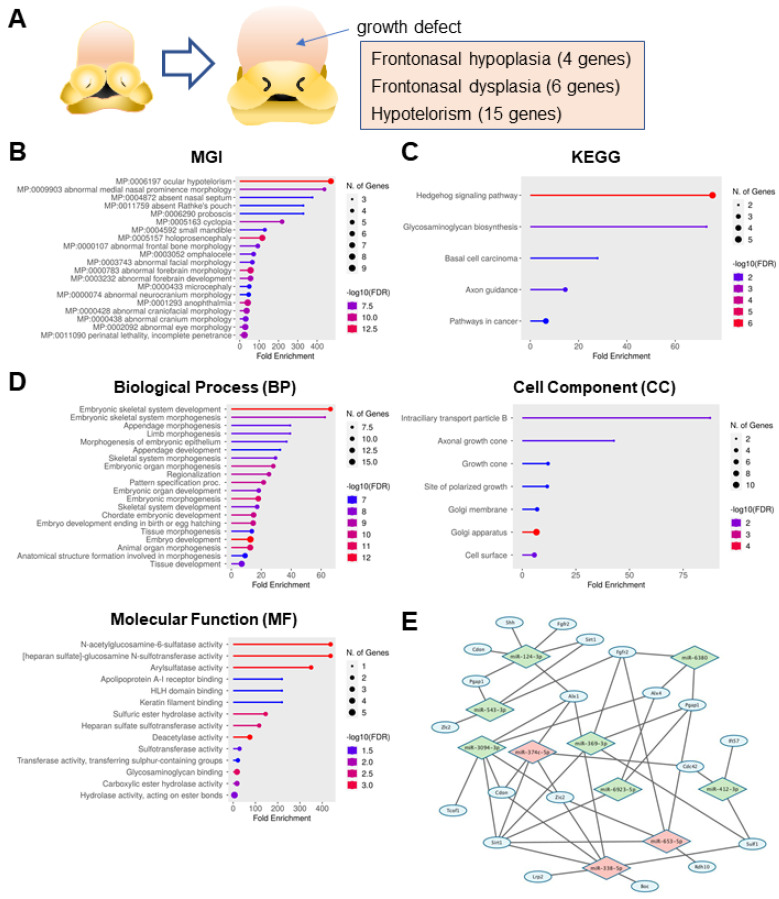
Bioinformatic characterization of genes and miRNAs related to frontonasal hypoplasia. (**A**) Schematic of the developing frontonasal region in mice at E9.5 and E10.5. The type of malformations and the number of mouse genes related to these malformations are shown. (**B**–**D**) Lollipop graphs for (**B**) MGI MP, (**C**) KEGG, and (**D**) GO analysis for biological process (BP), cell component (CC), and molecular function (MF). Circle size indicates the number of genes involved. Color code represents −log^10^ false discovery rate (FDR); low (blue) to high (red). (**E**) Visualization of integration with frontonasal hypoplasia-related genes and the predicted microRNA. Diamond (light green and pink) represents the predicted miRNAs; the top 3 miRNAs are highlighted in pink. Blue circle represents genes related to frontonasal malformations.

To collect information on mouse genes related to frontonasal malformations, we conducted a search of the MGI database using the terms ‘frontonasal hypoplasia’, ‘frontonasal dysplasia’, and ‘hypotelorism’, and then confirmed this information with the referenced literature. As a result, a total of 25 genes related to frontonasal malformations (4 genes in frontonasal hypoplasia, 6 genes in frontonasal dysplasia, and 15 genes in hypotelorism) were identified (Figure 1A and Table 1). An MGI mouse phenotype (MP) analysis showed that these genetic mutations were also often involved in ocular hypotelorism (MP:0006197), abnormal medial nasal prominence morphology (MP:0009903), and absent nasal septum (MP:0004872) (Figure 1B and Table 2). Next, we grouped these genes by cellular function and pathway with the KEGG pathway analysis and found that the genes were the most enriched with hedgehog signaling and glycosaminoglycan biosynthesis (Figure 1C and Table 2). Next, we conducted a GO analysis to identify the common biological processes (BPs), cell components (CCs), and molecular functions (MFs) of these genes. We found that the embryonic skeletal system development and embryonic skeletal system morphogenesis were the most enriched with the genes related to frontonasal malformations in BP, the most enriched in terms of the intraciliary transport particle B in CC, and N-acetylglucosamine-6-sulfatase activity and heparan sulfate-glucosamine N-sulfotransferase activity in MF (Figure 1D and Table 2).

Next, we analyzed miRNA target gene regulation using miRTarbase, miRanda, PITA, and TargetScan, and found that miR-338-5p potentially regulates the expression of *Boc*, *Lrp2*, *Alx1*, *Sulf1*, *Sirt1*, *Cdon*, and *Zic2*; miR-653-5p potentially regulates the expression of *Rdh10*, *Zic2*, *Pgap1*, *Sirt1*, and *Fgfr2*; and miR-374c-5p potentially regulates the expression of *Alx1*, *Cdc42*, *Sirt1*, *Cdon*, and *Zic2* (Figure 1E and Table 3).

### 3.2. Overexpression of miR-338-5p, miR-653-5p, and miR-374c-5p Inhibits Cell Proliferation and Suppresses Expression of Genes Related to Frontonasal Malformations in O9-1 Cells

**Figure 2:**
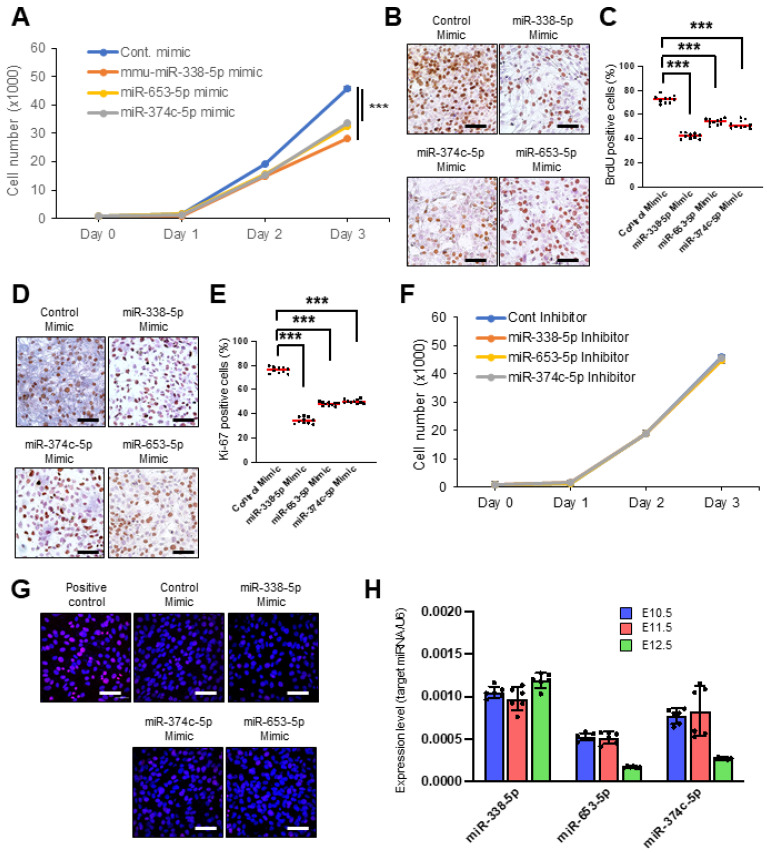
Effect of candidate miRNAs on cell proliferation. (**A**) Proliferation assays in O9-1 cells treated with the indicated miRNA mimic. *** *p* < 0.001. Each treatment group was compared to the control. (**B**) BrdU incorporation assays in O9-1 cells treated with the indicated miRNA mimic. Scale bar indicates 50 μm. (**C**) Quantification of BrdU incorporation assays in O9-1 cells treated with the indicated miRNA mimic. *** *p* < 0.001. Each treatment group was compared to the control. *n* = 10 per group. Red lines indicate median. (**D**) Immunocytochemical analysis for Ki-67 in O9-1 cells treated with the indicated miRNA mimic. Scale bars, 50 μm. (**E**) Quantification of immunocytochemical analysis for Ki-67 in O9-1 cells treated with the indicated miRNA mimic. *** *p* < 0.001. Each treatment group was compared to the control. *n* = 10 per group. (**F**) Cell proliferation assays in O9-1 cells treated with the indicated miRNA inhibitor. Each treatment group was compared to the control. *n* = 6 per group. (**G**) TUNEL assays in O9-1 cells treated with the indicated miRNA mimic or positive control. DAPI was used for nuclei staining. Scale bars, 50 μm. (**H**) Relative expression of the indicated miRNAs in the developing frontonasal region in C57BL/6J mice at E10.5 (blue), E11.5 (red), and E12.5 (green). *n* = 6 per group.

**Figure 3:**
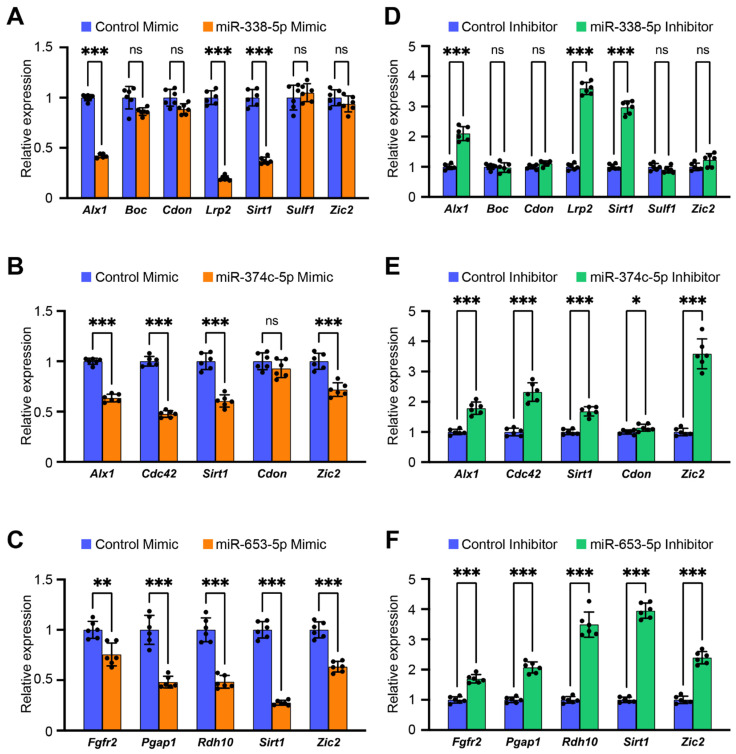
Effect of each miRNA mimic on the predicted target gene expression. (**A**–**C**) Quantitative RT-PCR for target genes in O9-1 cells treated with mimic for miR-338-5p, miR-653-5p, and miR-374c-5p for 24 h. ns, not significant. ** *p* < 0.01. *** *p* < 0.001. Each treatment group (orange) was compared with the negative control (blue). (**D**–**F**) Quantitative RT-PCR for target genes in O9-1 cells treated with inhibitor for miR-338-5p, miR-653-5p, and miR-374c-5p for 24 h. ns, not significant. * *p* < 0.05. *** *p* < 0.001. Each treatment group (green) was compared with the negative control (blue). *n* = 6 per group.

To test the functional significance of the candidate miRNAs (miR-338-5p, miR-653-5p, and miR-374c-5p) in cell proliferation, we performed cell proliferation assays with a specific mimic for each miRNA in O9-1 cells and found that the overexpression of these miRNAs significantly inhibited cell proliferation (Figure 2A). We confirmed these findings with Bromodeoxyuridine (BrdU) incorporation assays (Figure 2B,C) and immunocytochemical analyses for Ki-67 (Figure 2D,E). There was no apoptotic cell detected in the cells treated with these mimics in TUNEL assays (Figure 2F). Interestingly, we found that inhibitors for these miRNAs did not affect cell proliferation (Figure 2G), suggesting that expression of these miRNAs is relatively low in normal craniofacial development. Therefore, we measured expression of these miRNAs in the developing frontonasal primordium and found that these miRNAs are relatively low-expressed in the frontonasal primordium in C57BL6/J mice at embryonic day E10.5, E11.5, and E12.5 (Figure 2H). Next, to validate the predicted miRNA gene regulation, we conducted qRT-PCR analysis for the genes in O9-1 cells treated with each specific miRNA mimic and inhibitor. We found that the miR-338-5p mimic significantly downregulated expression of *Alx1*, *Lrp2*, and *Sirt1* in O9-1 cells (Figure 3A); the miR-374c-5p mimic significantly downregulated expression of *Alx1*, *Cdc42*, *Sirt1*, and *Zic2* (Figure 3B); and the miR-653-5p mimic significantly downregulated expression of *Fgfr2*, *Pgap1*, *Rdh10*, *Sirt1*, and *Zic2* (Figure 3C). To confirm the dose-dependent effect of the miRNAs on their target genes, the expression of the target genes was analyzed in presence of each miRNA inhibitor. We confirmed that the miR-338-5p inhibitor significantly upregulated expression of *Alx1*, *Lrp2*, and *Sirt1* (Figure 3D); the miR-374c-5p inhibitor significantly upregulated expression of *Alx1*, *Cdc42*, *Sirt1*, and *Zic2* (Figure 3E); and the miR-653-5p inhibitor significantly upregulated expression of *Fgfr2*, *Pgap1*, *Rdh10*, *Sirt1*, and *Zic2* (Figure 3F). Taken together, miR-338-5p, miR-653-5p, and miR-374c-5p can regulate the expression of genes related to frontonasal malformations in a dose-dependent manner.

## 4. Discussion

miRNAs play crucial roles in the survival of CNC cells during craniofacial development [19], and disruption of miRNA function in these cells results in frontonasal deformities in mice [10,11,12,20]. In this study, we found that overexpression of miR-338-5p, miR-653-5p, and miR-374c-5p inhibited cell proliferation in O9-1 cells through the regulation of genes related to frontonasal malformations. Interestingly, a recent study shows that overexpression of miR-338-5p is related to cleft palate [18]. Therefore, miR-338-5p may be one of the causative miRNAs related to a wide variety of craniofacial developmental defects. The role of miR-653-5p and miR-374c-5p remains largely unknown in both normal development and birth defects. These miRNAs may be upregulated, and therefore the genes crucial for normal development and functions will be downregulated under certain pathological conditions (e.g., cancers and birth defects). For instance, a recent study shows that exosomal miR-653-5p derived from mesenchymal stem cells suppresses laryngeal papilloma progression [21]. In addition, miR-653-5p plays a role in cell proliferation in various cancer cells, such as ovarian cancer [22], papillary thyroid carcinoma [23], breast cancer [24,25,26], gastric cancer [27], and lung cancer [28,29]. Although miR-374c-5p is less well characterized, recent studies show that miR-374c-5p secreted from mesenchymal stem cells inhibits cancer growth and metastasis formation by regulating the epithelial–mesenchymal transition [30,31].

The functions of each miRNA may differ by cell type and timing of expression. Therefore, this study is focused on cranial neural crest cells. In this study, we focused on cell proliferation to study early stages of frontonasal development. During these developmental stages (E10.5–E12.5), osteogenic differentiation is not much involved. In this study, we first curated genes related to frontonasal hypoplasia, frontonasal dysplasia, and hypotelorism and predicted the miRNAs that can regulate these genes. Our findings in regard to miRNA gene regulatory mechanisms will help us understand the potential causes of frontonasal anomalies. In addition, this set of genes will be useful to compare it with genes related to other craniofacial anomalies in order to study spaciotemporal mechanisms in craniofacial development. One of the limitations of this study is that, although we validated the results from bioinformatics with cell culture experiments, these miRNA functions should be evaluated in mouse models with frontonasal anomalies.

## Figures and Tables

**Table 1:** Genes related to frontonasal malformations.

Gene Symbol	References (PMID)	Chromosome	Description
**Frontonasal hypoplasia (4 genes)**			
*Bmp4*	24785830	14	bone morphogenetic protein 4
*Cdc42*	28326341	4	cell division cycle 42
*Ndst1*	16020517	18	N-deacetylase/N-sulfotransferase (heparan glucosaminyl) 1
*Rdh10*	17473173	1	retinol dehydrogenase 10 (all-trans)
**Frontonasal dysplasia (6 genes)**			
*Alx1*	35127681	10	ALX homeobox 1
*Alx3*	19409524	3	aristaless-like homeobox 3
*Alx4*	25673119	2	aristaless-like homeobox 4
*Fgfr2*	11274405	7	fibroblast growth factor receptor 2
*Ndst3*	18385133	3	N-deacetylase/N-sulfotransferase (heparan glucosaminyl) 3
*Tcof1*	16938878	18	treacle ribosome biogenesis factor 1
**Hypotelorism (15 genes)**			
*Boc*	21183473	16	biregional cell adhesion molecule-related/downregulated by oncogenes (Cdon) binding protein
*Cdon*	21183473	9	cell adhesion molecule-related/downregulated by oncogenes
*Disp1*	15269168	1	dispatched RND transporter family member 1
*Ift27*	25446516	15	intraflagellar transport 27
*Ift57*	17027958	16	intraflagellar transport 57
*Lrp2*	26107939	2	low-density lipoprotein receptor-related protein 2
*Nosip*	25546391	7	nitric oxide synthase-interacting protein
*Pgap1*	10529425	1	post-GPI attachment to proteins 1
*Shh* and *Sulf1* and *Sulf2*	18213582	5	sonic hedgehog
		1	sulfatase 1
		2	sulfatase 2
*Shh* and *Six3*	18694563	17	sine oculis-related homeobox 3
*Sirt1*	28273169	10	sirtuin 1
*Wdr11*	29263200	7	WD repeat domain 11
*Zic2*	29992973	14	zinc finger protein of the cerebellum 2

PMID: PubMed identifier.

**Table 2:** Enrichment analysis for genes related to frontonasal malformations.

Enrichment FDR	Gene Number	Pathway Genes	Pathway	Genes
**MGI enrichment**				
1.70 × 10^−15^	7	13	MP:0006197 ocular hypotelorism	*Ift27*, *Sirt1*, *Disp1*, *Ift57*, *Wdr11*, *Zic2*, *Pgap1*
1.01 × 10^−8^	4	8	MP:0009903 abnormal medial nasal prominence morphology	*Tcof1*, *Rdh10*, *Wdr11*, *Pgap1*
2.83 × 10^−6^	3	7	MP:0004872 absent nasal septum	*Tcof1*, *Rdh10*, *Lrp2*
3.62 × 10^−6^	3	8	MP:0011759 absent Rathke’s pouch	*Bmp4*, *Fgfr2*, *Pgap1*
3.62 × 10^−6^	3	8	MP:0006290 proboscis	*Ndst1*, *Zic2*, *Pgap1*
3.84 × 10^−9^	5	20	MP:0005163 cyclopia	*Shh*, *Disp1*, *Wdr11*, *Zic2*, *Pgap1*
1.50 × 10^−6^	4	27	MP:0004592 small mandible	*Ift27*, *Tcof1*, *Wdr11*, *Pgap1*
1.01 × 10^−12^	8	60	MP:0005157 holoprosencephaly	*Shh*, *Lrp2*, *Disp1*, *Cdon*, *Six3*, *Wdr11*, *Zic2*, *Pgap1*
1.64 × 10^−7^	5	47	MP:0000107 abnormal frontal bone morphology	*Shh*, *Tcof1*, *Disp1*, *Fgfr2*, *Alx4*
5.49 × 10^−7^	5	61	MP:0003052 omphalocele	*Ift27*, *Bmp4*, *Lrp2*, *Alx4*, *Ndst1*
8.23 × 10^−7^	5	67	MP:0003743 abnormal facial morphology	*Disp1*, *Fgfr2*, *Wdr11*, *Ndst1*, *Pgap1*
9.08 × 10^−12^	9	141	MP:0000783 abnormal forebrain morphology	*Shh*, *Tcof1*, *Rdh10*, *Lrp2*, *Disp1*, *Alx1*, *Cdon*, *Wdr11*, *Pgap1*
4.79 × 10^−9^	7	110	MP:0003232 abnormal forebrain development	*Bmp4*, *Tcof1*, *Lrp2*, *Six3*, *Ndst1*, *Zic2*, *Pgap1*
2.87 × 10^−6^	5	89	MP:0000433 microcephaly	*Shh*, *Tcof1*, *Wdr11*, *Zic2*, *Pgap1*
3.62 × 10^−6^	5	95	MP:0000074 abnormal neurocranium morphology	*Shh*, *Tcof1*, *Disp1*, *Fgfr2*, *Alx4*
1.08 × 10^−10^	9	191	MP:0001293 anophthalmia	*Shh*, *Bmp4*, *Tcof1*, *Lrp2*, *Six3*, *Wdr11*, *Ndst1*, *Zic2*, *Pgap1*
4.79 × 10^−9^	8	196	MP:0000428 abnormal craniofacial morphology	*Shh*, *Sirt1*, *Tcof1*, *Rdh10*, *Fgfr2*, *Cdon*, *Six3*, *Wdr11*
1.64 × 10^−7^	7	200	MP:0000438 abnormal cranium morphology	*Shh*, *Cdc42*, *Tcof1*, *Fgfr2*, *Six3*, *Ndst1*, *Pgap1*
2.05 × 10^−8^	8	246	MP:0002092 abnormal eye morphology	*Shh*, *Sirt1*, *Bmp4*, *Tcof1*, *Rdh10*, *Lrp2*, *Ndst1*, *Pgap1*
6.25 × 10^−9^	9	324	MP:0011090 perinatal lethality, incomplete penetrance	*Shh*, *Ift27*, *Sirt1*, *Bmp4*, *Lrp2*, *Cdon*, *Alx4*, *Wdr11*, *Pgap1*
**KEGG enrichment**				
2.80 × 10^−7^	5	58	Hedgehog signaling pathway	*Shh*, *Boc*, *Lrp2*, *Disp1*, *Cdon*
8.51 × 10^−3^	2	24	Glycosaminoglycan biosynthesis	*Ndst3*, *Ndst1*
2.93 × 10^−2^	2	63	Basal cell carcinoma	*Shh*, *Bmp4*
1.87 × 10^−2^	3	181	Axon guidance	*Shh*, *Cdc42*, *Boc*
3.11 × 10^−2^	4	542	Pathways in cancer	*Shh*, *Cdc42*, *Bmp4*, *Fgfr2*
**GO enrichment (BP)**				
1.64 × 10^−13^	10	132	Embryonic skeletal system development	*Shh*, *Sulf2*, *Alx3*, *Sulf1*, *Bmp4*, *Rdh10*, *Fgfr2*, *Alx1*, *Alx4*, *Ndst1*
2.84 × 10^−9^	7	98	Embryonic skeletal system morphogenesis	*Alx3*, *Bmp4*, *Rdh10*, *Fgfr2*, *Alx1*, *Alx4*, *Ndst1*
4.78 × 10^−8^	7	156	Appendage morphogenesis	*Shh*, *Alx3*, *Sulf1*, *Bmp4*, *Rdh10*, *Alx1*, *Alx4*
4.78 × 10^−8^	7	156	Limb morphogenesis	*Shh*, *Alx3*, *Sulf1*, *Bmp4*, *Rdh10*, *Alx1*, *Alx4*
6.96 × 10^−8^	7	166	Morphogenesis of embryonic epithelium	*Shh*, *Sulf1*, *Bmp4*, *Rdh10*, *Lrp2*, *Ift57*, *Alx1*
1.35 × 10^−7^	7	188	Appendage development	*Shh*, *Alx3*, *Sulf1*, *Bmp4*, *Rdh10*, *Alx1*, *Alx4*
2.49 × 10^−8^	8	237	Skeletal system morphogenesis	*Alx3*, *Sulf1*, *Bmp4*, *Rdh10*, *Fgfr2*, *Alx1*, *Alx4*, *Ndst1*
2.20 × 10^−10^	10	312	Embryonic organ morphogenesis	*Shh*, *Alx3*, *Bmp4*, *Rdh10*, *Fgfr2*, *Ift57*, *Alx1*, *Six3*, *Alx4*, *Ndst1*
5.79 × 10^−10^	10	348	Regionalization	*Shh*, *Bmp4*, *Lrp2*, *Disp1*, *Ift57*, *Alx1*, *Cdon*, *Six3*, *Alx4*, *Pgap1*
2.20 × 10^−10^	11	446	Pattern specification proc.	*Shh*, *Alx3*, *Bmp4*, *Lrp2*, *Disp1*, *Ift57*, *Alx1*, *Cdon*, *Six3*, *Alx4*, *Pgap1*
1.08 × 10^−8^	10	478	Embryonic organ development	*Shh*, *Alx3*, *Bmp4*, *Rdh10*, *Fgfr2*, *Ift57*, *Alx1*, *Six3*, *Alx4*, *Ndst1*
1.76 × 10^−11^	13	632	Embryonic morphogenesis	*Shh*, *Alx3*, *Sulf1*, *Bmp4*, *Rdh10*, *Lrp2*, *Fgfr2*, *Ift57*, *Alx1*, *Cdon*, *Six3*, *Alx4*, *Ndst1*
1.80 × 10^−8^	10	508	Skeletal system development	*Shh*, *Sulf2*, *Alx3*, *Sulf1*, *Bmp4*, *Rdh10*, *Fgfr2*, *Alx1*, *Alx4*, *Ndst1*
1.61 × 10^−10^	13	765	Chordate embryonic development	*Shh*, *Sulf2*, *Alx3*, *Sulf1*, *Bmp4*, *Tcof1*, *Rdh10*, *Lrp2*, *Fgfr2*, *Ift57*, *Alx1*, *Alx4*, *Ndst1*
1.75 × 10^−10^	13	781	Embryo development ending in birth or egg hatching	*Shh*, *Sulf2*, *Alx3*, *Sulf1*, *Bmp4*, *Tcof1*, *Rdh10*, *Lrp2*, *Fgfr2*, *Ift57*, *Alx1*, *Alx4*, *Ndst1*
1.11 × 10^−7^	10	639	Tissue morphogenesis	*Shh*, *Cdc42*, *Sulf1*, *Bmp4*, *Rdh10*, *Lrp2*, *Fgfr2*, *Ift57*, *Alx1*, *Six3*
1.64 × 10^−13^	17	1169	Embryo development	*Shh*, *Sulf2*, *Alx3*, *Sulf1*, *Bmp4*, *Tcof1*, *Rdh10*, *Lrp2*, *Disp1*, *Fgfr2*, *Ift57*, *Alx1*, *Cdon*, *Six3*, *Alx4*, *Ndst1*, *Pgap1*
1.76 × 10^−11^	15	1041	Animal organ morphogenesis	*Shh*, *Cdc42*, *Sulf2*, *Alx3*, *Sulf1*, *Bmp4*, *Rdh10*, *Lrp2*, *Fgfr2*, *Ift57*, *Alx1*, *Cdon*, *Six3*, *Alx4*, *Ndst1*
1.24 × 10^−7^	12	1144	Anatomical structure formation involved in morphogenesis	*Shh*, *Cdc42*, *Sulf1*, *Sirt1*, *Bmp4*, *Tcof1*, *Rdh10*, *Lrp2*, *Fgfr2*, *Ift57*, *Alx1*, *Cdon*
2.49 × 10^−8^	15	1900	Tissue development	*Shh*, *Cdc42*, *Sulf2*, *Sulf1*, *Sirt1*, *Bmp4*, *Tcof1*, *Rdh10*, *Lrp2*, *Fgfr2*, *Ift57*, *Alx1*, *Cdon*, *Six3*, *Alx4*
**GO enrichment (CC)**				
1.50 × 10^−2^	2	20	Intraciliary transport particle B	*Ift27*, *Ift57*
2.56 × 10^−2^	2	41	Axonal growth cone	*Boc*, *Lrp2*
3.78 × 10^−2^	3	218	Growth cone	*Sirt1*, *Boc*, *Lrp2*
3.78 × 10^−2^	3	225	Site of polarized growth	*Sirt1*, *Boc*, *Lrp2*
3.78 × 10^−2^	4	502	Golgi membrane	*Cdc42*, *Ift27*, *Ndst3*, *Ndst1*
2.48 × 10^−5^	11	1449	Golgi apparatus	*Shh*, *Cdc42*, *Sulf2*, *Ift27*, *Sulf1*, *Lrp2*, *Ndst3*, *Ift57*, *Alx1*, *Wdr11*, *Ndst1*
1.60 × 10^−2^	6	926	Cell surface	*Shh*, *Sulf2*, *Sulf1*, *Lrp2*, *Fgfr2*, *Cdon*
**GO enrichment (MF)**				
3.98 × 10^−4^	2	4	N-acetylglucosamine-6-sulfatase activity	*Sulf2*, *Sulf1*
3.98 × 10^−4^	2	4	[heparan sulfate]-glucosamine N-sulfotransferase activity	*Ndst3*, *Ndst1*
4.44 × 10^−4^	2	5	Arylsulfatase activity	*Sulf2*, *Sulf1*
4.63 × 10^−2^	1	4	Apolipoprotein A-I receptor binding	*Cdc42*
4.63 × 10^−2^	1	4	HLH domain binding	*Sirt1*
4.63 × 10^−2^	1	4	Keratin filament binding	*Sirt1*
1.94 × 10^−3^	2	12	Sulfuric ester hydrolase activity	*Sulf2*, *Sulf1*
2.64 × 10^−3^	2	15	Heparan sulfate sulfotransferase activity	*Ndst3*, *Ndst1*
3.98 × 10^−4^	3	35	Deacetylase activity	*Sirt1*, *Ndst3*, *Ndst1*
3.12 × 10^−2^	2	61	Sulfotransferase activity	*Ndst3*, *Ndst1*
4.63 × 10^−2^	2	82	Transferase activity, transferring sulphur-containing groups	*Ndst3*, *Ndst1*
1.94 × 10^−3^	4	202	Glycosaminoglycan binding	*Shh*, *Sulf2*, *Sulf1*, *Bmp4*
1.20 × 10^−2^	3	152	Carboxylic ester hydrolase activity	*Ndst3*, *Ndst1*, *Pgap1*
2.11 × 10^−2^	5	742	Hydrolase activity, acting on ester bonds	*Sulf2*, *Sulf1*, *Ndst3*, *Ndst1*, *Pgap1*

**Table 3:** miRNA prediction for regulating genes related to frontonasal malformations.

miRNA Family	q-Value Bonferroni	q-Value FDR B and H	Hit Count in Query List	Target Gene
miR-338-5p	5.66 × 10^−5^	1.26 × 10^−4^	7	*Alx1*, *Boc*, *Cdon*, *Lrp2*, *Sirt1*, *Sulf1*, *Zic2*
miR-653-5p	8.68 × 10^−4^	9.98 × 10^−3^	5	*Fgfr2*, *Pgap1*, *Rdh10*, *Sirt1*, *Zic2*
miR-374c-5p	2.95 × 10^−2^	1.55 × 10^−2^	5	*Alx1*, *Cdc42*, *Cdon*, *Sirt1*, *Zic2*
miR-543-3p	1.15 × 10^−2^	2.64 × 10^−2^	4	*Fgfr2*, *Zlc2*, *Sirt1*, *Pgap1*
miR-124-3p	5.85 × 10^−2^	2.64 × 10^−2^	6	*Alx1*, *Cdon*, *Fgfr2*, *Shh*, *Sirt1*, *Pgap1*
miR-6923-5p	1.17 × 10^−1^	3.38 × 10^−2^	3	*Alx4*, *Pgap1*, *Sirt1*
miR-3094-3p	1.33 × 10^−1^	3.49 × 10^−2^	6	*Alx1*, *Alx4*, *Cdon*, *Sirt1*, *Tcof1*, *Zic2*
miR-6380	2.30 × 10^−1^	3.77 × 10^−2^	3	*Alx4*, *Fgfr2*, *Pgap1*
miR-369-3p	2.81 × 10^−1^	4.17 × 10^−2^	4	*Fgfr2*, *Pgap1*, *Sirt1*, *Sulf1*
miR-412-3p	3.16 × 10^−1^	4.34 × 10^−2^	3	*Cdc42*, *Ift57*, *Sulf1*

B and H: Benjamini–Hochberg.

## Data Availability

Data are contained within the article and Supplementary Materials.

## Supplementary Materials

The following supporting information can be downloaded at: https://www.mdpi.com/article/10.3390/jdb12030019/s1, Table S1: Primer list used in this study.
