# Supplementary material for: Genes Related to Frontonasal Malformations Are Regulated by miR-338-5p, miR-653-5p, and miR-374-5p in O9-1 Cells
Source: J Dev Biol. 2024 Jul 6;12(3):19. doi: 10.3390/jdb12030019 (PMC11270360; doi:10.3390/jdb12030019)
Supplement: Supplementary file 1 [file jdb-12-00019-s001.zip › jdb-2971344-supplementary.pdf]

Table S1. Primer list used in this study

Gene	Forward Sequence
Alx1	CCAGGGTCCAGGTTTGGTT
Alx3	TGGCTTTGCGAACAGACCTGAC
Alx4	GGACGGTAGCCTCAAGCTCCA
Bmp4	GCCGAGCCAACACTGTGAGGA
Boc	TCCAAGACGGACTCCTATGAGC
Cdc42	ATGTGAAAGAAAAGTGGGTGCC
Cdon	TGAAGGACAGCCTGCCATGCTT
Disp1	GGTCAGACGATCACCATGAGAG
Fgfr2	GTCTCCGAGTATGAGTTGCCAG
Ift27	CACCAAGTGGTTGGAGAAGGTC
Ift57	GAGCAGCCTCAAGAATACGACG
Lrp2	CCAATGGACTCACTCTGGACCT
Ndst1	GCGAACAGAACCTGCCAAAGTC
Ndst3	TGGACCAACCTGCGTCTTCAGA
Nosip	GGAGTACATCCTGCACCAGAAG
Pgap1	TTCTCCACTCAGTCAACCTCTG
Rdh10	TGGTCAACTGCCACGCACACTT
Shh	GGATGAGGAAAACACGGGAGCA
Sirt1	GGAGCAGATTAGTAAGCGGCTTG
Six3	CCCACACAAGTAGGCAACTGGT
Sulf1	GGTCAAGGAACTGTGCCAGCAA
Sulf2	CGTCTCAAGCACAAAGGCTCCA
Tcof1	GAGGACTCAGACAGCAGTAGTG
Wdr11	CACCATCTCACAACCTGAAGAGC
Zic2	ACGCTCCGAGAACCTCAAGATC

Reverse Sequence
TTCAAATGCGTGTCCGTTGG
CGGAGATGTCATAGGCTGTAGTG
CCTGTCTCCTTCACACTGAGGT
GATGCTGCTGAGGTTGAAGAGG
GCCAGAAATGGTCCAGTCGTCA
GATGCGTTCATAGCAGCACAC
CCTGGAGGAATCCGTAAGCACA
CCTCCTGCTGAAGCAAACACTAC
CCACTGCTTCAGCCATGACTAC
CCTTGACTCAGTGCCCATACCT
GCACACTTGTTCTCCGTAACCAG
GAATGGAAGGCAGTGCTGATGAC
CTCCTTCAAGCAGTTGGCACCA
AGACCAAATGTCTCTGTGGCGTT
GAAGCCTCTAACTTGGTCCTGG
ATGCTGCACTTGTTTTCCTCG
CCTCAACTCCAGCAGTGCTGAA
GGATGAGGAAAACACGGGAGCA
GTTACTGCCACAGGAACTAGAGG
CTGAGCCGTGCGTGGGGCAG
AGGTTGCGTGCATTCTGACGGA
GCTGCACGTATCGTTGTTCTGC
GTGTTCACAGCCTGGACTCCTT
GCCTTACTCTCTGCCTCCTGTA
GGCTTATCTGAGGTGTGGACATG
